# Uncommon Incidental Diagnosis of Lipomatous Hypertrophy of the Interatrial Septum

**DOI:** 10.7759/cureus.43589

**Published:** 2023-08-16

**Authors:** Sheridan Rose, Caleb Hood, Emily Custer, Kortni McCall

**Affiliations:** 1 Department of Internal Medicine, Edward Via College of Osteopathic Medicine, Auburn, USA; 2 Department of Internal Medicine, University of Alabama at Birmingham School of Medicine, Birmingham, USA; 3 Department of Internal Medicine, Huntsville Hospital, Huntsville, USA

**Keywords:** interatrial septum, asymptomatic cardiac lesion, cardiac mechanical obstruction, benign cardiac mass, lipomatous hypertrophy of interatrial septum

## Abstract

Lipomatous hypertrophy of the interatrial septum (LHIS) is a benign non-neoplastic cardiac lesion that previously has not been readily described, but with the increasing usage of computed tomography and echocardiography, this is now becoming a more well-characterized incidental finding. This case highlights an incidental finding of LHIS while a patient was undergoing treatment for a routine gastrointestinal bleed.

## Introduction

Current literature suggests that approximately 75% of primary cardiac lesions are benign and most are classified as a myxoma [1]. The remaining 25% represent the malignant neoplasms, advanced secondary metastasis is more common than primary malignant cardiac neoplasms. Metastasis from pleural mesothelioma, melanoma, and lung adenocarcinoma constitute most malignant cardiac neoplasms [2]. There is a separate benign cardiac lesion that is commonly mistaken for a heart tumor, lipomatous hypertrophy of the interatrial septum (LHIS). LHIS is defined as a non-encapsulated mass of fatty tissue that infiltrates the atrial septum [3]. In this case we present a patient who complained of symptoms of a routine gastrointestinal bleed and during the course of treatment was diagnosed incidentally with LHIS.

## Case presentation

A chronically ill non-ambulatory 56-year-old Caucasian woman presented to the emergency department with a one-week history of persistent nausea, vomiting, and generalized weakness. Past medical history was significant for chronic obstructive pulmonary disease, gastroesophageal reflux disease, and chronic anemia.

Workup in the emergency department noted the patient to be afebrile, with systolic blood pressures in the mid-100s and stable, along with a heart rate consistently in the 90s. Initial laboratory testing revealed a hemoglobin level of 8.4 g/dL and a stool sample positive for occult blood (Table 1). All x-ray imaging showed no acute abnormalities. The patient on a physical exam was noted to be extremely weak with skin pale in color.

**Table 1:** Initial blood workup results

Blood Test	Result	Reference Range
Sodium	133 mmol/L	135-145 mmol/L
Potassium	3.6 mmol/L	3.5-5.1 mmol/L
Chloride	99 mmol/L	98-107 mmol/L
Bicarbonate	31 mmol/L	22-30 mmol/L
Blood Urea Nitrogen	15 mg/dL	7-17 mg/dL
Creatinine	1.0 mg/dL	0.52-1.04 mg/dL
Glucose	92 mg/dL	74-106 mg/dL
Bilirubin, total	0.7 mg/dL	0.1- 1.2 mg/dL
Aspartate Transaminase (AST)	12 U/L	8-33 U/L
Alanine Transaminase (ALT)	8 U/L	4-36 U/L
Alkaline Phosphatase	137 U/L	44- 147 U/L
Hemoglobin	8.4 g/dL	12.0- 15.0 g/dL
Hematocrit	25.7 %	38.6- 49.2 %
Platelets	204 x 10^3^/mL	150- 450 x 10^3^/mL
White Blood Cells	4.0 x 10^3^/mL	4.5- 11.0 x 10^3^/mL

**Figure 1:**
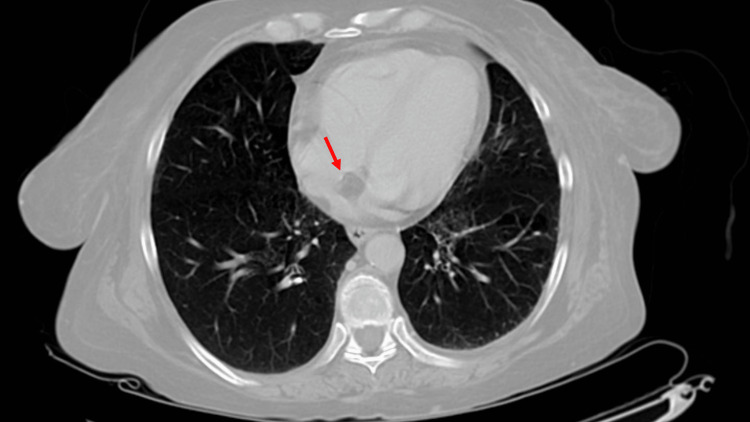
Smooth, non-enhancing, well-marginated 14.9 mm mass visualized on non-contrast computed tomography, characteristically sparing the fossa ovalis and maintaining an elongated shape

Abdominal ultrasound aimed to evaluate an etiology for the nausea and vomiting. Ultrasound noted a large cystic area in the epigastrium. Subsequently, a computed tomography (CT) scan was ordered to evaluate the abdomen for epigastric cystic pathology; the results showed a 14.90 mm lipomatous collection of the interatrial septum in the heart (Figure 1). The cardiology service was consulted and on recommendations from the cardiologist, a transthoracic echocardiogram (TTE) was performed to evaluate the heart function. The TTE confirmed the lipomatous hypertrophy of the interatrial septum, along with a left ventricular ejection fraction of 65%, indicating normal systolic heart function. Further recommendations from the cardiologist were to have a cardiac CT scan in the outpatient setting to surveil the lesion. The patient was discharged on hospital day seven to a rehabilitation facility following the resolution of gastrointestinal bleeding.

The patient was readmitted to the hospital 11 days post-discharge for altered mental status. On admission, the patient was noted to have a high sensitivity cardiac troponin level of 3726 ng/L and a normal electrocardiogram, the status of chest pain could not be ascertained due to the patient's altered mental status. The patient was diagnosed with a non-ST elevated myocardial infarction. A repeat TTE in the setting of ischemia revealed a preserved left ventricular ejection fraction. A left heart catheterization was scheduled to evaluate for coronary artery disease; however, it was deferred. The cardiology service felt there was no new cardiac tissue damage due to troponins trending down in the setting of preserved ejection fraction and due to the patient's altered mental status preventing them from complying with procedure instructions. The patient was stabilized, discharged to home, and scheduled for outpatient follow-up with a cardiologist.

## Discussion

LHIS is a benign non-neoplastic cardiac lesion and was first characterized in an autopsy study done by Prior in 1964 [4]. While the exact etiology of LHIS is unclear, it is thought to develop from the limbus of the fossa ovalis, a structure that is representative of the embryological septum secundum. During embryogenesis of the heart, the developing atria require an in-folding process that results in mesodermal tissue being drawn into the wall of the atrial septum and becoming entrapped [3]. Throughout the lifetime in an unelucidated process, the entrapped adipose tissue undergoes hyperplasia and expands resulting in a large mass within the atrial septum. Histologically LHIS differs from a lipoma in that there will be entrapped atrial myocytes, along with both white and brown fat [5]. Morphologically, LHIS presents as a non-encapsulated excessive epicardial fat deposition, whereas lipomas classically present as encapsulated, round, homogenous, and do not infiltrate the myocardial fibers [6].

Clinically LHIS can present with symptoms on a spectrum that ranges from silent to fatal atrial cardiac arrhythmias. The increased usage of advanced imaging techniques allows for the identification of asymptomatic cardiac masses at an improved rate. A prospective study found that LHIS was identified in 2.2% of patients who underwent a multislice CT scan of the thorax [7]. The differential diagnosis of LHIS can be accomplished using a variety of multimodality imaging; the most utilized are echocardiography, cardiac CT, or magnetic resonance imaging. As highlighted in our case, CT and transthoracic echocardiography are sufficient for making a definitive diagnosis, eliminating the need for biopsy and histological examination. The lesion maintains a non-enhancing elongated oval or "dumbbell shape” with smooth boundaries on non-contrast-enhanced CT. The characteristic shape is due to being derived from the upper portion of the atrial septum with fat accumulation cephalad and caudad to the fossa ovalis, sparing this structure’s involvement [8]. This is in contrast with other cardiac lesions such as a myxoma which arise within proximity of the fossa ovalis.

While our case highlights the asymptomatic nature of LHIS, this condition can be associated with increased instances of atrial arrhythmias. The pathogenesis of arrhythmias is correlated to the degree of disruption of right atrial myocyte architecture. The zone of fat infiltration can result in both an ectopic focus for impulse generation and a re-entry circuit for cardiac impulses [5]. These manifest mainly in atrial fibrillation, atrial premature complexes, supraventricular arrhythmias, and ectopic and junctional rhythms [6]. In addition to arrhythmias, the size and localization of the lesion may cause obstructive symptoms by bulging into the right atria resulting in right-sided heart failure or superior vena cava syndrome. In both arrhythmias and obstruction, there is a role for surgical resection of the lesion to eliminate symptoms. The mechanism by which resection resolves arrhythmia is unclear. However, this has been directly demonstrated by Dickerson et al. in which they presented the case of a patient with LHIS and symptomatic atrial flutter which was resolved following resection of the lesion [9]. In the case of asymptomatic lesions, yearly imaging to monitor for enlarging size and the presence of new-onset symptoms is sufficient.

## Conclusions

In conclusion, our case demonstrates the incidental discovery of asymptomatic lipomatous hypertrophy of the interatrial septum. While this condition is uncommon, it must be considered as a differential diagnosis for any right atrial cardiac mass, as well as an etiology for arrhythmia and obstructive symptoms. Definitive diagnosis can be accomplished using multimodality imaging techniques, negating the need for invasive biopsy procedures. Yearly surveillance imaging is appropriate for patients with asymptomatic lesions to monitor for growth progression. Surgical resection can play a role in resolution of symptoms for those patients with arrhythmias or signs of cardiac outlet obstructions. 
